# Short-latency prepulse inhibition of the trigeminal blink reflex

**DOI:** 10.3389/fnins.2024.1357368

**Published:** 2024-05-22

**Authors:** Megumi Shingaki, Yasushi Itoh, Bayasgalan Borgil, Tetsuo Kida, Koji Inui

**Affiliations:** Department of Functioning and Disability, Institute for Developmental Research, Aichi Developmental Disability Center, Kasugai, Japan

**Keywords:** GABA receptor, IPSP, prepulse inhibition, R1, startle reflex

## Abstract

Prepulse inhibition (PPI) is a well-established phenomenon wherein a weak sensory stimulus attenuates the startle reflex triggered by a subsequent strong stimulus. Within the circuit, variations in target responses observed for PPI paradigms represent prepulse-induced excitability changes. However, little is known about the mechanism of PPI. Here, we focused on short-latency PPI of the trigeminal blink reflex R1 signal with an oligosynaptic reflex arc through the principal sensory trigeminal nucleus and the facial nucleus. As the facial nucleus is facilitatory to any input, R1 PPI is the phenomenon in the former nucleus. Considering that GABAergic modulation may be involved in PPI, this study investigated whether the PPI mechanism includes GABA-A equivalent inhibition, which peaks at approximately 30 ms in humans. In 12 healthy volunteers, the reflex was elicited by electrical stimulation of the supraorbital nerve, and recorded at the ipsilateral lower eyelid by accelerometer. Stimulus intensity was 1.5 times the R1 threshold for test stimulus and 0.9 times for the prepulse. The prepulse–test interval (PTI) was 5–150 ms. Results showed significant inhibition at 40-and 80–150-ms PTIs but not at 20-, 30-, 50-, 60-, and 70-ms PTIs, yielding two distinct inhibitions of different time scales. This corresponds well to the early and late components of inhibitory post synaptic potentials by GABA-A and GABA-B receptor activation. Thus, the data support the contribution of inhibitory post synaptic potentials elicited by the prepulse to the observed PPI. As inhibitory function-related diseases may impair the different inhibition components to varying degrees, methods deconvoluting each inhibitory component contribution are of clinical importance.

## Introduction

Cortical neural circuits usually consist of both excitatory pyramidal neurons and inhibitory interneurons, with the balance between these elements crucial for controlling circuit outputs. However, understanding the mechanism of inhibitory interneurons, especially in humans, remains limited, primarily due to the lack of non-invasive methods for observing inhibitory postsynaptic potentials (IPSPs). Additionally, inhibitory interneurons have highly complex morphologies, function, and interactions within neural circuits (Mody and Pearce, 2004). Usually, voltage clamping is required to observe IPSPs but it is technically difficult and time-consuming to reveal interneuron functions through excitatory neuron interactions. Therefore, current research techniques are yet to fully elucidate inhibitory interneuron mechanisms. However, inhibitory interneuron function is clinically important as neural inhibition impairment may contribute to certain diseases, such as schizophrenia (Marín, 2012) and autism (Cellot and Cherubini, 2014). Unfortunately, there are few testing methods for inhibitory functions in humans. As there are many types of inhibitory interneurons, with diseases likely to impair each specific neuron or receptor functions differently, it is ideal to investigate the function of each neuron type. However, this far exceeds current technical capabilities, particularly as, in humans, the excitation/inhibition balance at the single neuron level cannot be directly observed. One possible approach to circumvent this is to evaluate the output behavior of the system.

There are a few indirect methods to observe excitability changes of a circuit, including auditory P50 gating (Adler et al., 1982; Takeuchi et al., 2022), paired pulse suppression (Kimura, 1973; Badawy et al., 2012), and prepulse inhibition (PPI) of the startle reflex (Braff, 2010). Each method uses a preceding stimulus to change neural circuit excitability, influencing the response to a second stimulus. Of these, PPI is unique in that the first stimulus is weak, insufficient to elicit a target response. PPI also benefits from the exclusion of complications relating to a strong first stimulus, such as transmitter depletion. PPI is usually observed using startle reflexes elicited by a sound. That is, an initial weak sound suppresses the startle reflex normally elicited by a subsequent loud sound. Patients with schizophrenia are known to have impaired PPI (Grillon et al., 1992; Mena et al., 2016) as well as model animals (Marín, 2012). PPI is generally indexed by startle reflex magnitude, measured in humans by recording blinks using electromyography and in animals by quantifying whole-body movements associated with being startled (Gómez-Nieto et al., 2020). In animals, key areas involved in the PPI neural circuit for the acoustic startle reflex include the cochlear root neuron, caudal pontine reticular nucleus, and facial nucleus (Davis et al., 1982; Koch, 1999; López et al., 1999; Zheng and Schmid, 2023). However, translating this to humans is challenging due to the pathway’s complexity and incomplete knowledge of the mechanisms involved in PPI. Considering this, we recently developed a method using the trigeminal blink reflex R1 component to observe PPI (Inui et al., 2023). The reflex elicited by electrical stimulation of the supraorbital nerve (SON) has multiple components, including an early and ipsilateral component (R1) and a later bilateral component (R2). The R1 component is sharp, occurring within the first 10 to 20 ms of the blink reflex, and is formed by a two-synapse reflex circuit via the trigeminal principal sensory nucleus and facial nerve nucleus (May and Warren, 2021). Thus, it has some advantages over the conventional paradigm due to reflex stability and circuit simplicity. By manipulating the prepulse–test interval (PTI), inhibition mechanisms may be deconvoluted. Another advantage to this method is that early inhibition may be explored due to the sharp and brief nature of electrical stimulation. Recently, clear PPI of the R1 component was demonstrated using the principal trigeminal nucleus as the target site of inhibition (Inui et al., 2023).

As inhibitory interneurons in the brain use GABA as a transmitter, it is possible that GABAergic mechanisms are involved in PPI (Kodsi and Swerdlow, 1995; Yeomans et al., 2010; Inui et al., 2018). For example, PPI can occur at the first stage of the acoustic startle reflex in cochlear root neurons (Gómez-Nieto et al., 2014) that receive GABAergic inputs (Osen et al., 1991). Given that PPI has GABA-mediated components, variations in peak inhibition latency will then arise depending on receptor type IPSP time course. This may explain the multiple inhibition peaks in the PTI time axis (Inui et al., 2018). As the time course of IPSPs, GABA-mediated postsynaptic potentials, are known from patch-clamping recordings both in animals and humans (McCormick, 1989), it is possible to examine possible relationships between PPI inhibitory components and specific IPSPs. In the present study, we focused on early inhibition corresponding to early GABA-A inhibition peaking at approximately 30 ms (Kawaguchi, 1992), which enables correct nervous system function and precise information processing (Wehr and Zador, 2003) by outlasting the early excitatory post synaptic potentials (EPSPs) (Connors et al., 1988). For example, early inhibition is important to achieve millisecond precision timing in neural activity (Wehr and Zador, 2003). Thus, the R1 of the trigeminal blink reflex is considered beneficial to investigate short PTIs. In rodents, a GABA-A receptor antagonist was shown to reduce the acoustic startle reflex PPI at PTIs near the peak of inhibition at 10–100 ms, while a GABA-B receptor antagonist reduced PPI at only long PTIs (Yeomans et al., 2010). If inhibition by GABA receptors is a common mechanism of synaptic regulation, then the PPI of R1 may also behave similarly.

## Materials and methods

This study was approved in advance by the Ethics Committee of Aichi Developmental Disability Center, Kasugai, Japan (approval number: R04-09) and conducted in accordance with the Declaration of Helsinki. Written informed consent was obtained from all subjects. The study was performed on 12 healthy volunteers (5 females and 7 males) aged 34.3 ± 12.6 (ranging between 26 and 57) years. None of the subjects were treated for neurological or mental diseases or substance abuse in the last 2 years. Subjects were non-smokers and did not use hormone agents including contraceptives.

### Electrical stimulation

The method of electrical stimulation, recording of the blink reflex, and the procedure for analysis followed a recent study (Inui et al., 2023). To elicit blink reflexes, the right SON was stimulated with a square wave pulse of 0.5 ms using two disposable Ag/AgCl gel electrodes 10 mm in diameter (Biorode SDC-H, Vyaire Medical, Tokyo), one placed near the supraorbital foramen and the other approximately 3 cm above it. The current intensity was 1.5 times the R1 threshold for the test stimulus and 0.9 times for the prepulse. The R1 threshold was defined as the current at which R1 was elicited in 50% of stimulations. The stimulus frequency was 2 Hz.

### Recording of the blink reflex

Subjects were seated in a chair and instructed to gaze at a fixed point, 1.5 m ahead, with their eyes open. Blink reflexes were recorded from the right orbicularis muscles using a single-axis accelerometer (8 × 8 × 4 mm; MPS110, Medi Sens Inc., Tokyo, Japan) placed on the central part of the lower eyelid. The analoge filter was set at 1–250 Hz. Signals were amplified and stored in an EMG/EP measuring system (MEB-2300, Nihon Kohden, Tokyo) at a sampling rate of 10,000 Hz. The analysis window was 40 ms before to 160 ms after test stimulus onset.

### Experimental design

**Figure 1:**
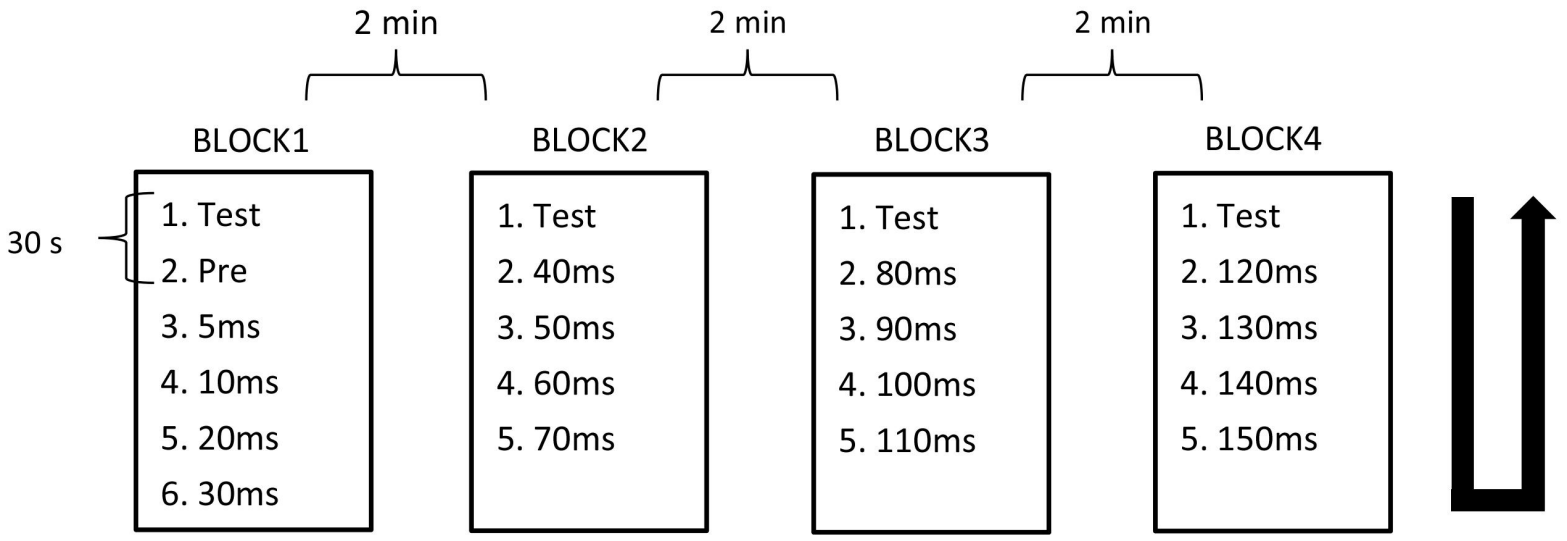
Block design. Recordings were divided into four blocks containing five or six conditions. Condition recordings were divided into 2 runs with 15 trials. The order of runs in each block was from top to bottom, then bottom to top as indicated by the U-shaped line.

The PPI paradigm employed used prepulse–test intervals (PTIs) of 5 ms and 10–150 ms in 10-ms steps. The recording of stimulus conditions was divided into four blocks with fixed combinations: PTIs of 5, 10, 20, and 30 ms in Block 1, 40, 50, 60, and 70 ms in Block 2, 80, 90, 100, and 110 ms in Block 3, and 120, 130, 140, and 150 ms in Block 4. In each block, there were five or six conditions including test alone, prepulse alone, and prepulse + test with four different PTIs. Block order was randomized across subjects. For each stimulus condition, 15 epochs were recorded per run, with either 12 or 10 runs conducted, and a 30-s interval between each run to accumulate a total of 30 epochs for each stimulus within a block. In a block, the order of runs followed an ascending then descending sequence, with the run for test-alone stimulation positioned as the first and last. The interval between blocks was 2 min. Figure 1 provides a schematic overview of the experimental design.

### Procedures of analyses

Each blink response was fully rectified and averaged across the block. Then, the pre-stimulus baseline subtracted and area under the curve (AUC) calculated at 15–40 ms and used as the R1 response magnitude. The degree of inhibition was calculated as the percentage of the response amplitude of a test + prepulse condition, relative to the test alone condition of the same block (%Amplitude). Significant differences in the degree of inhibition among 16 PTI conditions were analyzed using one-way repeated measures analysis of variance (ANOVA). Significance of the prepulse effect on each test + prepulse response was assessed using 95% confidence intervals of the %Amplitude; with confidence intervals not overlapping 100, determined to be significant. The effect of sex on the degree of inhibition was assessed by two-way ANOVA with sex and PTI as variables. Relationship between age and PPI was assessed by simple linear correlation analysis and partial correlation analysis controlling for sex. The effect of block order on the amplitude of the test alone response was examined by one-way repeated measures ANOVA. Statistical significance was set at *p* values less than 0.05. For statistical analyses, SPSS version 24 was used. Data are expressed as the mean ± standard deviation.

## Results

**Figure 2:**
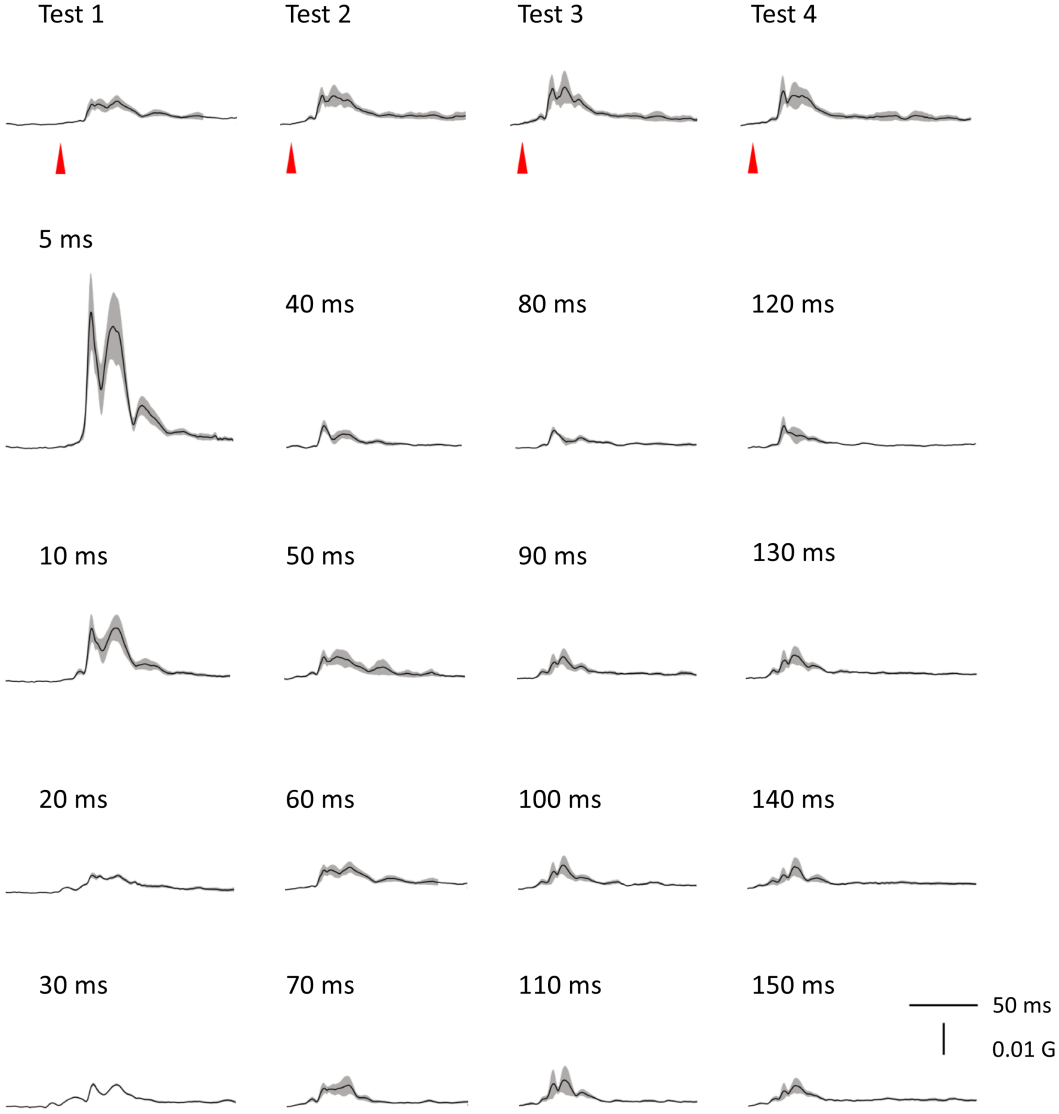
Grand-averaged waveforms. Averaged waveforms across subjects for all blocks are shown. Shaded areas indicate ± SE.

**Figure 3:**
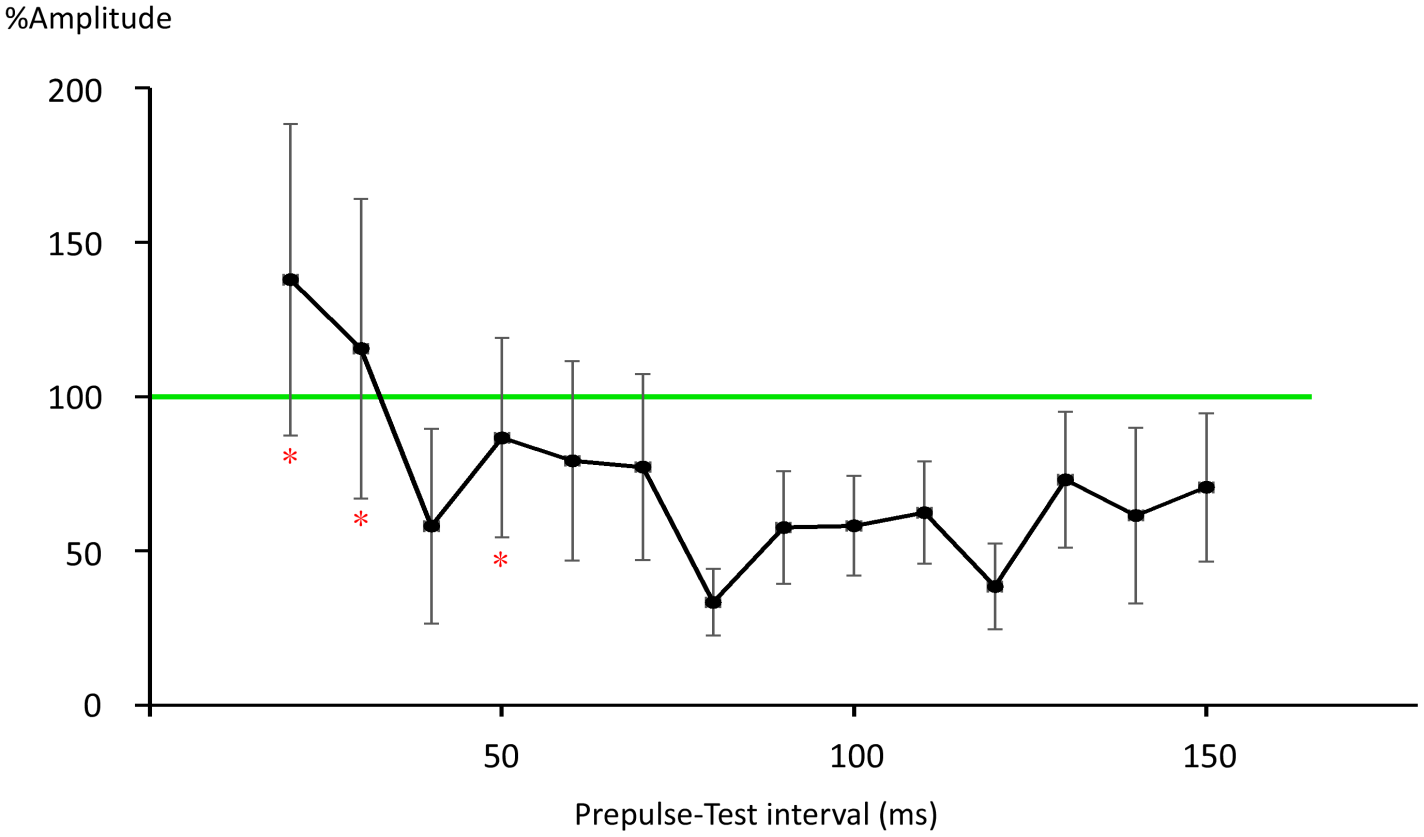
Plots of %Amplitude against the prepulse–test interval. Vertical bars indicate 95% confidence intervals. The vertical axis shows R1 amplitude at each prepulse–test interval relative to amplitude of the test-alone condition (%Amplitude). The green line indicates baseline %Amplitude without prepulse effects. Vertical bars indicate the 95% confidence interval. PTI conditions with vertical bars not overlapping the green line were judged as significant. *significant difference from the 40-ms condition (*p* < 0.05).

There was no significant difference in age (*p* = 0.61) between females (40.4 ± 12.4) and males (43.7 ± 9.7). In all subjects, SON electrical stimulation elicited a clear R1 signal. The test stimulus intensity (1.5 times R1 threshold) was 5.9 ± 2.0 mA. As recordings were divided into four blocks, the order effect on the test alone response was examined using one-way repeated measures ANOVA. Results showed that block order did not significantly affect the test alone amplitude (*F*_3,33_ = 1.92, *p* = 0.15, η^2^ = 0.15). With a weak prepulse (0.9 times R1 threshold), PPI of R1 was observed. Variation of PTIs between 5 and 150 ms modulated R1 response amplitudes to the test stimulation at certain PTIs. Figure 2 shows grand-averaged waveforms for each condition. The ANOVA results indicated a significant difference in %Amplitude among 16 PTIs (*F*_15, 165_ = 25.5, *p* = 2.4 × 10^−35^). Further analysis revealed significant R1 inhibition by the prepulse at PTIs of 5, 10, 40, and 80–150 ms (Tables 1, 2). Notably, significant R1 inhibition by the prepulse occurred at two distinct time scales, approximately 40 ms and longer than 80 ms. Figure 3 shows plots of the %Amplitude against PTI. Paired t-tests confirmed a discrete inhibition peak for the PTI of 40 ms, with significantly smaller %Amplitude compared to PTIs of 5, 10, 20, 30, and 50 ms (*p* < 0.033, uncorrected for multiple comparisons). The observed R1 inhibition by the prepulse at specific PTIs suggests the involvement of inhibitory mechanisms in modulating the trigeminal blink reflex. This finding implies that certain PTIs may effectively suppress the startle reflex.

**Table 1:** The %Amplitude at each prepulse–test interval (PTI) in each subject.

PTI (ms)	Subject number											
	1	2	3	4	5	6	7	8	9	10	11	12
BLOCK1												
5	402.4	521.2	689.9	1005.0	351.6	1007.7	84.2	1433.0	1007.7	245.4	453.6	466.2
10	256.5	393.3	225.1	373.9	198.6	437.0	54.6	530.8	437.0	131.0	289.8	240.8
20	128.2	212.9	93.5	184.0	82.4	76.4	8.2	253.7	76.4	171.5	265.0	101.9
30	200.0	224.0	114.7	191.7	110.1	55.4	1.8	206.3	55.4	31.1	139.4	56.0
BLOCK2												
40	19.6	132.5	63.0	15.9	69.0	17.7	33.9	150.9	39.3	−2.0	114.7	41.1
50	103.8	83.9	79.3	38.9	146.7	97.1	40.8	208.0	66.9	24.6	94.1	55.0
60	75.7	72.9	51.3	46.0	105.6	145.9	35.3	200.8	73.2	22.1	43.7	77.3
70	121.1	62.1	71.7	96.3	138.2	106.9	7.6	159.7	53.3	34.9	25.3	47.9
BLOCK3												
80	17.8	26.7	46.3	47.5	42.8	29.3	14.1	55.5	23.4	62.3	9.4	24.8
90	58.2	49.7	64.7	47.7	61.1	26.1	41.2	134.5	52.7	22.6	77.3	55.0
100	69.9	40.4	92.2	57.1	60.1	20.8	41.6	113.4	58.4	52.5	31.9	58.7
110	86.7	73.1	94.6	22.6	62.6	14.6	63.7	89.4	61.6	44.6	48.1	86.7
BLOCK4												
120	18.9	29.8	20.2	54.6	20.4	1.8	43.5	61.1	68.0	56.8	23.4	62.6
130	73.3	91.0	101.3	83.9	38.3	89.8	19.8	129.0	113.9	46.2	27.4	62.6
140	86.2	93.4	92.7	51.2	22.6	32.7	25.3	62.2	171.8	47.8	5.9	45.2
150	71.9	148.1	63.2	92.7	33.3	91.0	19.8	100.9	103.9	41.0	33.2	48.2

**Table 2:** The average %Amplitude and 95% confidence intervals at each PTI.

PTI (ms)	%Amplitude (SD)	Median	95% Confidence interval	
			Lower limit	Upper limit
BLOCK1				
5	639.0 (395.2)	493.7	387.9	890.1
10	297.4 (139.8)	273.2	208.5	386.2
20	137.8 (79.4)	115.1	87.4	188.3
30	115.5 (76.5)	112.4	66.9	164.1
BLOCK2				
40	58.0 (49.7)	40.2	26.4	89.6
50	86.6 (50.9)	81.6	54.3	118.9
60	79.2 (50.8)	73.1	46.9	111.4
70	77.1 (47.4)	66.9	47.0	107.2
BLOCK3				
80	33.3 (17.1)	28.0	22.5	44.2
90	57.6 (28.7)	53.9	39.3	75.8
100	58.1 (25.3)	57.7	42.0	74.2
110	62.4 (26.0)	63.2	45.8	78.9
BLOCK4				
120	38.4 (21.9)	36.7	24.5	52.3
130	73.0 (34.7)	78.6	51.0	95.1
140	61.4 (44.8)	49.5	33.0	89.9
150	70.6 (37.8)	67.6	46.6	94.6

Age correlation to the degree of inhibition was assessed by correlation analysis. No significant correlation was found for any PTI (|*r*| < 0.33, *p* > 0.30). Partial correlation analysis controlling for sex did not influence the results (*p* > 0.34). The effect of sex on %Amplitude was evaluated by two-way ANOVA using sex and PTI as variables. Results showed that sex was not a significant factor in influencing the degree of inhibition (*F*_1,10_ = 0.07, *p* = 0.80, *η*^2^ = 0.007) despite the slightly greater overall %Amplitude for females (126.5 ± 24.4%) than males (118.1 ± 20.7%). The interaction between sex and PTI was not statistically significant (*F*_15,1_ = 1.51, *p* = 0.11, *η*^2^ = 0.13).

## Discussion

**Figure 4:**
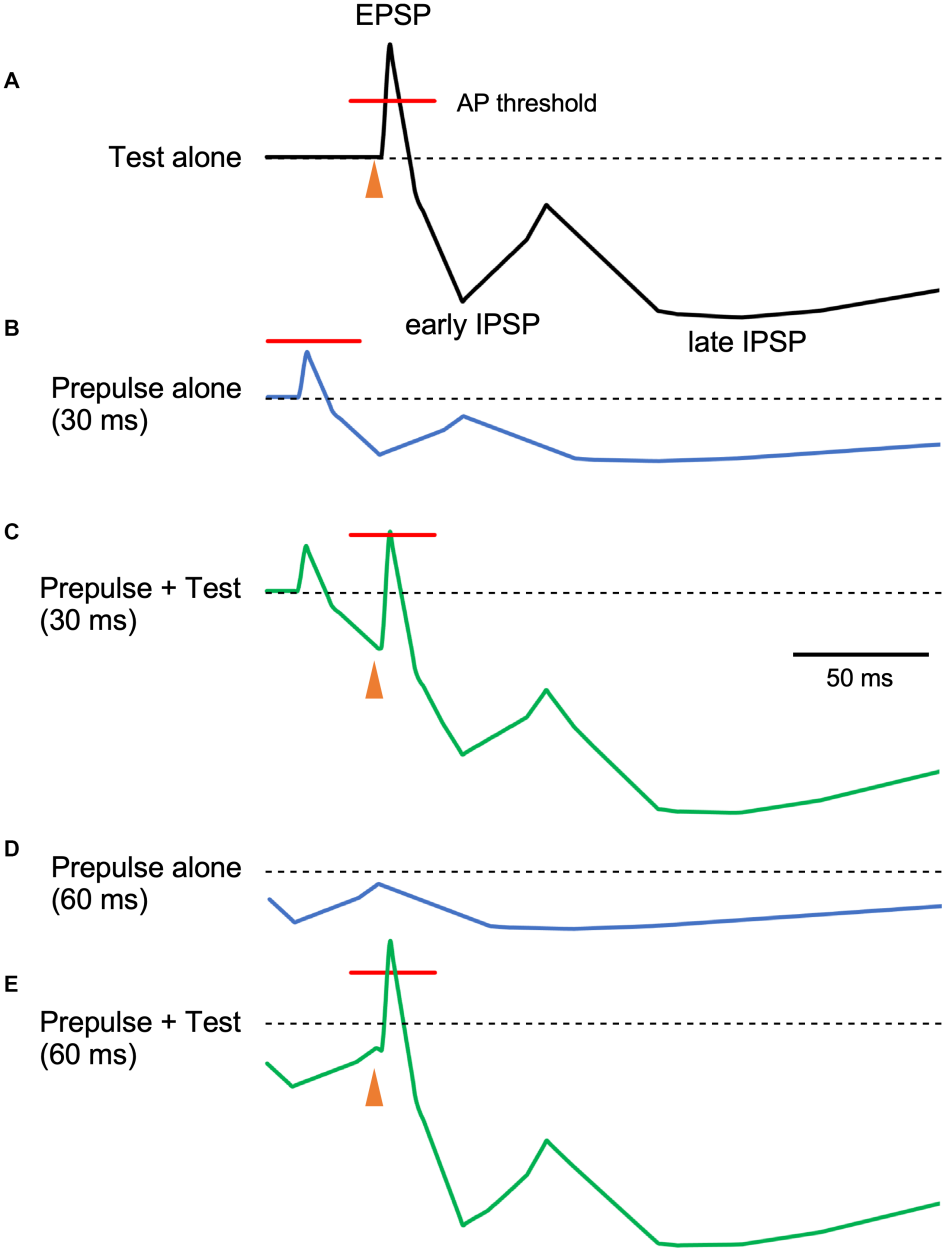
The relationship between prepulse–test interval and inhibitory postsynaptic potentials. Schematic representation of neuron membrane potential. Depolarization due to excitatory inputs is quickly canceled out by slight delayed inhibitory postsynaptic potentials (IPSPs), inducing sharp depolarization **(A)**. When the membrane potential exceeds the firing threshold (indicated red lines), action potentials (APs) occur within the narrow window. As compared to EPSPs, IPSPs have a long duration with several components. In this figure, early and late components peaking at around 30 and 140 ms, respectively are shown. As the stimulus intensity of the prepulse is just below the R1 threshold, EPSPs due to the prepulse do not exceed the firing threshold **(B)**. Here, the magnitude of postsynaptic potentials of the prepulse is set as 40% of the test alone response. When both stimuli are delivered at a prepulse–test interval of 30 ms, the sharp EPSP due to the test stimulus is reduced by temporally overlapping early IPSPSs due to the prepulse **(C)**, reducing firing frequency. On the other hand, at the PTI of 60 ms **(D)**, hyperpolarization due to the prepulse at around 60 ms is small and the resultant decrease in EPSP is modest **(E)**. As such, IPSP time course may be indirectly evaluated by manipulating PTI. Filled arrowheads, test stimulus. The postsynaptic potential time course was based on intracellular recordings from human brain slices by McCormick (1989).

In the present study, we evaluated whether PPI of the R1 trigeminal blink reflex has an early component consistent with early GABA-A inhibition. Results show significant R1 suppression by the prepulses at 40 ms and longer than 80 ms, suggesting at least two inhibitory mechanisms are involved. As prepulses with 0.9 times the threshold were used, passive mechanisms, such as a depressing synapse, are unlikely. Given the threshold intensity similarity between EPSPs and IPSPs for individual neurons or even smaller for IPSPs (Kawaguchi, 1992; Isaacson and Scanziani, 2011), prepulse-induced IPSPs may reduce the action potential rate of target neurons either by hyperpolarization-induced reductions in firing chance or by narrowing the time window for firing (Figure 4). Test-induced EPSPs and concomitant test-induced IPSPs with a slight time gap sharpens test-evoked depolarization and the addition of an IPSP (prepulse-induced) with a similar time course further makes the window for generating action potentials narrow. The present results are consistent with the hypothesis that there is a short-latency R1 PPI with a time course reflecting GABA-A induced IPSPs. Additionally, these results suggest several mechanisms with different time scales contribute to R1 PPI. Although other mechanisms are known to affect pyramidal neuron–pyramidal neuron transmission, those due to strong activation caused by the first stimulus, such as transmitter depletion, are unlikely to contribute to the present results. For example, paired-pulse or frequency-dependent synaptic inhibition depends on EPSP magnitude in presynaptic pyramidal neurons (Thomson et al., 1993) and input frequency (Thomson, 1997).

Multiple mechanisms may be involved in R1 PPI. As fundamental circuit inhibitory mechanisms, two IPSPs are well known (Connors et al., 1988). The early IPSP overlaps EPSPs of a pyramidal neuron temporarily, and is attributed to action through GABA-A receptors (Connors et al., 1988; McCormick, 1989; Kawaguchi, 1992). The peak latency of early hyperpolarization induced by GABA-A receptor activation is reported as 10–30 ms for the rat sensorimotor cortex (Avoli, 1986), 29.4 ms for rat frontal cortex (Kawaguchi, 1992), 28 ms for human temporal cortex (McCormick, 1989), and earlier for current injection stimulation of interneurons (Tamás et al., 1997). Thus, the observed early inhibition at 40 ms corresponds well with early GABA-A inhibition. Due to response augmentation at earlier PTIs, the inhibition at 20-or 30-ms PTI was unclear in this study. However, facilitation at 20 and 30 ms was not significant, implying both facilitation and inhibition at these PTIs.

The late IPSP, a function of GABA-B receptors, follows with an onset latency of approximately 50 ms, peaking at around 140–190 ms in rats and rabbits (Satou et al., 1982; Connors et al., 1988; Davies et al., 1990; Kawaguchi, 1992). In humans, the late GABA-B mediated IPSP reportedly peaks at 135 ms (McCormick, 1989). These values are consistent with the longer than 80 ms PTI inhibition times observed, as well as our previous study reporting peak R1 inhibition at 140 ms (Inui et al., 2023). Therefore, the biphasic inhibition time course observed in the present study matches well with the early and late IPSPs common across mammals (Connors et al., 1988) and brain areas (Packer and Yuste, 2011). For later IPSPs, a canonical component also arises from Marcinotti cells (Fino et al., 2013; Karnani et al., 2014), potentially contributing to inhibition at long PTIs. However, the IPSP latency due to Marcinotti cells appears too long to constitute a major component of the present late inhibition. For example, Silberberg and Markram (2007) reported the latency of such IPSPs to be 240 ms, with these IPSPs also sensitive to the number of action potentials in the presynaptic pyramidal neurons. That is, stronger inputs induce IPSPs with shorter latencies and greater amplitudes. Therefore, this cell group is unlikely to strongly contribute to inhibition within PTIs of 150 ms. However, the %Amplitude–PTI curve in Figure 3 shows more than two peaks, implying the existence of additional inhibitory mechanisms other than the traditional early and late components. Further research is needed to clarify this.

Neural inhibition abnormalities are considered important in certain neuropsychiatric disorders including epilepsy (McCormick and Contreras, 2001; Noebels, 2003), schizophrenia (Marín, 2012), and autism (Cellot and Cherubini, 2014). Despite many years of study, a detailed PPI mechanism is yet to be achieved; R1 PPI observation may clarify mechanisms involving GABA receptors or inhibitory interneurons. If clinical conditions are related to abnormalities of specific interneurons or receptors, then PPI changes may occur at specific PTIs. The simplicity of the R1 circuit facilitates study, involving the trigeminal principal nucleus and facial nucleus, with the former demonstrated as the R1 PPI target site (Inui et al., 2023). However, this does not imply that inhibitory control is simple. Rather, it is complicated by the involvement of many interneuron types. The present paradigm is useful for deconvoluting certain components. Early PTI inhibition appears particularly important in neuropsychiatric disorders as early inhibition allows accurate signal processing (Wehr and Zador, 2003). In fact, GABA-A receptor-mediated inhibitory dysfunction is thought involved in these diseases (Dienel and Lewis, 2019; Cherubini et al., 2022). The experimental method used in this study is non-invasive, and allows for easy response observation without placing additional burdens on the examinee. As a tool for examining short-latency inhibition, the R1 PPI paradigm has merit for the precise stimulus timing and short inter-trial interval. A long inter-trial interval is required to elicit the startle reflex, and under such conditions a prepulse markedly facilitates the blink reflex at short PTIs, obscuring early inhibition (Inui et al., 2023). However, before applying the present method in clinical studies, effects of age and sex require clarification using a larger sample size. Although such effects were not observed in this study, the sample size was insufficient to exclude such possibilities. Such biases are not remote as previous paired pulse or prepulse inhibition studies reported these factors significantly affecting the degree of inhibition (Swerdlow et al., 1993; Kumari et al., 2010; Inui et al., 2024). The significant potentiation of the response at 5-and 10-ms PTIs are consistent with a previous study showing paired pulse stimulation to the supraorbital nerve augmented R1 at these PTIs (Kimura and Harada, 1976). In fact, Kimura recommended the use of double pulses at a 5-ms interval to elicit clear R1 signals (Kimura, 1975). Marked potentiation in the present study is likely due to the use of 1.5 times the R1 threshold to elicit the test response, which was smaller than the maximum response. Although the exact mechanism of potentiation remains unclear, it is believed that the facial nucleus is significant in enhancing responses. This is based on previous findings that the nucleus always facilitatory to inputs, regardless of their source (Inui et al., 2023). That study also showed that potentiation did not occur in muscle at PTIs longer than 40 ms. Whether the same is true for shorter PTIs requires future evaluation.

## Data availability statement

The original contributions presented in the study are included in the article/supplementary material, further inquiries can be directed to the corresponding author.

## Ethics statement

The studies involving humans were approved by Ethics Committee of Aichi Developmental Disability Center, Kasugai, Japan (approval number: R04-09). The studies were conducted in accordance with the local legislation and institutional requirements. The participants provided their written informed consent to participate in this study.

## Author contributions

MS: Conceptualization, Data curation, Formal analysis, Investigation, Project administration, Validation, Writing – original draft. YI: Data curation, Methodology, Writing – review & editing. BB: Data curation, Writing – review & editing. TK: Data curation, Writing – review & editing. KI: Data curation, Writing – review & editing, Conceptualization, Writing – original draft.
